# Total Synthesis of Annonaceous Acetogenins Belonging to the Non-Adjacent Bis-THF and Non-Adjacent THF-THP Sub-Classes 

**DOI:** 10.3390/molecules15010460

**Published:** 2010-01-21

**Authors:** Ian B. Spurr, Richard C. D. Brown

**Affiliations:** The School of Chemistry, The University of Southampton, Highfield, Southampton SO17 1BJ, UK; E-Mail: ibs@soton.ac.uk (I.B.S.)

**Keywords:** annonaceous acetogenins, non-adjacent bis-THF, tetrahydrofuran, tetrahydropyran, asymmetric synthesis, 1,2-diols, epoxides, cytotoxic natural products, anti-tumour

## Abstract

The synthesis of the subgroups of acetogenins containing non-adjacent bis-THF and non-adjacent THF-THP core units is reviewed. Specifically, total syntheses of gigantecin, 4-deoxygigantecin, *cis*-sylvaticin, squamostatin-C, squamostatin-D, sylvaticin and mucocin are discussed.

## 1. Introduction

**Figure 1:**
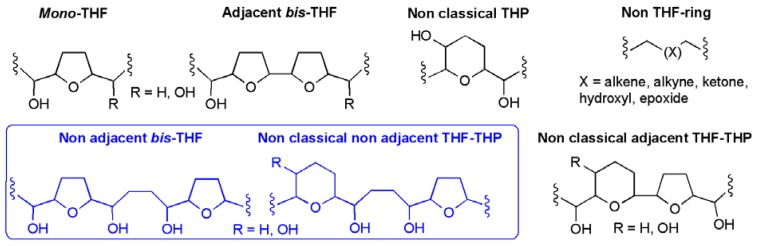
Classification of annonaceous acetogenins.

The annonaceous acetogenins are a group of natural products isolated from the Annonaceae (or custard apple) family of plants [1,2,3,4,5,6,7,8,9]. Purification of the extracts from the seeds, leaves and twigs of these plants typically yields acetogenins as waxy low-melting substances containing C_32_ or C_34_ unbranched fatty acid backbones. At first inspection, many of these acetogenins appear to be structurally similar, sharing a number of common features including 2,5-disubstituted tetrahydrofuran rings (and less commonly tetrahydropyran rings), secondary alcohol groups, and a butenolide or lactone ring. However, structural and stereochemical isomerism coupled with the occurrence of other functional groups means that more than 400 different annonaceous acetogenins have been identified (Figure 1) [2].

Due to the large number annonaceous acetogenins that have been isolated and characterised, a system of classification has been introduced, which groups them according to their core structures (Figure 1). In this review attention will focus only on the total synthesis of acetogenins belonging to the non-adjacent bis-THF and non-adjacent THF-THP classes, where the cyclic ether systems are separated by four carbon atoms.

**Figure 2:**
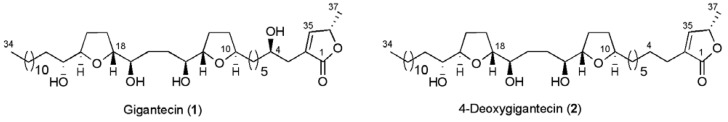
Structures of non-adjacent bis-THF acetogenins gigantecin (**1**) and 4-deoxygigantecin (**2**).

The core classes can be broken down further into sub classes by the nature of the γ-lactone, but commonly a methyl substituted α,β-unsaturated γ-lactone (butenolide) is present with or without hydroxylation at C4 in the linking chain (Figure 2). Standardised numbering for annonaceous acetogenins begins at the lactone carbonyl carbon (C1), the numbering continues down the alkyl backbone (up to C34 or C36) with the remaining lactone carbons numbered as shown. This numbering will be used throughout this review.

**Figure 3:**
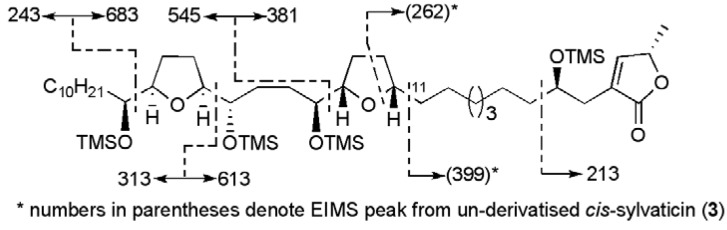
EIMS fragmentation for derivatised and un-derivatised *cis*-sylvaticin (**3**) [13].

Due to their waxy physical nature, structural assignment of acetogenins by X-ray crystallography has rarely been accomplished. One exception relevant to the current review is gigantecin (**1**), which yielded an X-ray structure, thus providing confirmation of its relative stereochemistry [10]. Absolute stereochemistry could be deduced on the basis that all of the known acetogenins possess the *S* configuration within their butenolide ring system (C36 in gigantecin). More typically a combination of various MS and NMR techniques are employed for structural determination, including the Mosher ester method for determining absolute stereochemistry of secondary alcohols [11,12]. Thus, molecular weight is established by ESI mass spectrometry, while THF/THP ring and hydroxyl positions on the alkyl chain are found through careful analysis of mass spectrometry fragmentation data (Figure 3).

**Figure 4:**
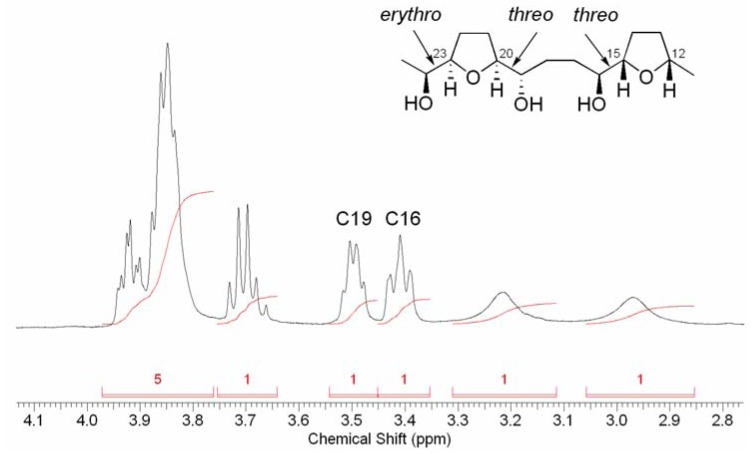
Partial ^1^H-NMR spectrum of *cis*-sylvaticin (**3**) showing carbinol methine resonances for *threo* configured hydroxyalkyl THFs (C15/C16 and C19/C20).

Comparisons of ^1^H- and ^13^C-NMR data from synthetic THF/THP cores with isolated acetogenins have been used to determine relative stereochemistry [11,14,15,16,17], although some caution should be exercised because certain groups of diastereoisomers containing cores with local symmetry cannot be differentiated [11,18,19]. However, none of the structures discussed herein possess local symmetry due to the absence of the hydroxyl group between the central THF ring and the butenolide (C9 of gigantecin, C11 of *cis*-sylvaticin). An important observation from the ^1^H-NMR spectra of THF-diol portion in acetogenins is that carbinol methine protons of hydroxyalkyl THFs possessing *threo* relative configurations resonate around δ 3.4–3.6 ppm, while carbinol methine protons of the corresponding *erythro* compounds resonate at ~δ 3.8 ppm [14]. Therefore, from the ^1^H-NMR spectrum of *cis*-sylvaticin (**3**) it can be deduced that two of the carbinol groups flanking the THF rings had *threo* relative configurations and the other one had an *erythro* relationship, although their exact positions could not be assigned purely from the ^1^H-NMR spectrum of *cis*-sylvaticin (Figure 4) [13].

**Scheme 1:**
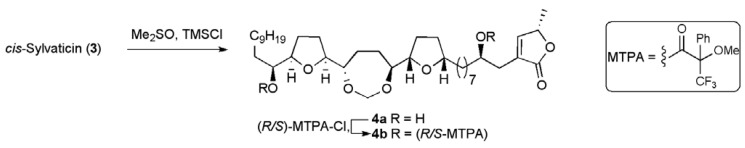
Synthesis of derivatives to enable stereochemical determination for *cis*-sylvaticin.

The relative relationship between C16 and C19 in *cis*-sylvaticin was established through ^1^H-NMR analysis of the formylidene acetal **4a** (Scheme 1). Subsequent formation and NMR analysis of the C4,C24 di-Mosher ester derivatives **4b** then provided the absolute stereochemistry at these stereogenic centres, and therefore in the non-adjacent bis-THF core [13]. In other non-adjacent bis-THF acetogenins, absolute stereochemistry was tentatively assigned on the basis of analogy with known structures, awaiting confirmation through total syntheses.

The annonaceous acetogenins have many interesting biological effects, including *in vivo* antitumor, anti-parasitic, pesticidal, antimalarial and antibacterial activities [2,3,4,5,6,7,8,9]. Most notably Annonaceous acetogenins act as cytotoxic anti-tumour agents, some of them possessing exceptional cytotoxicity in several human tumor cell lines (ED_50_ > 10^–12^ μg/mL) [20,21]. Interestingly, anti-tumor activity can extend to multi-drug resistant (MDR) cancer cell lines [22,23]. Cytotoxic and anti-tumour properties of Annonaceous acetogenins are linked to their potent inhibition of mitochondrial complex I, and NADH oxidases found in cancer cells [24,25,26,27]. In fact some acetogenins rank among the most potent known inhibitors of complex I [28,29,30,31].

## 2. Total Syntheses of Non-Adjacent Bis-THF Acetogenins

Annonaceous acetogenins have provoked considerable interest from synthetic chemists and many total syntheses have been reported in the literature. These syntheses have been reviewed previously by several groups [2,32,33,34,35,36], including a substantial recent review by Li *et al.* in 2008 [37]. Due to the significant number of total syntheses of the Non-Adjacent compounds, we felt that a detailed and up to date review dedicated to this acetogenin sub-classes would provide a valuable resource for researchers working with acetogenins. Herein total syntheses of squamostatin-D, 4-deoxygigantecin, gigantecin, squamostatin-C, *cis*-sylvaticin, sylvaticin and mucocin will be discussed.

Before examining these total syntheses in detail it is important to appreciate the structural relationships between these different natural products synthesised. Gigantecin, squamostatin-C, *cis*-sylvaticin and sylvaticin are isomeric structures: gigantecin and squamostatin-C differ in the position of the non-Adjacent bis-THF unit along the carbon backbone, while *cis*-sylvaticin, sylvaticin and squamostatin-C are diastereoisomers. 4-Deoxygigantecin is isomeric with squamostatin-D, the two structures differing only in the position of the non-adjacent bis-THF core and the configuration of their outermost carbinol groups (C22 and C24 respectively). In order to approach the total synthesis of these molecules, methods for the stereocontrolled formation of *trans* and *cis* configured THF diols will be required, which also allow for control of the vicinal oxygen functionalities. In addition, the correct connectivity between the two cyclic ether systems needs to be established. The successful realization of these objectives will be discussed within the context of the total syntheses of non-adjacent bis-THF acetogenins. Incomplete syntheses, the preparation of analogues and model studies will not be reviewed here.

**Figure 5:**
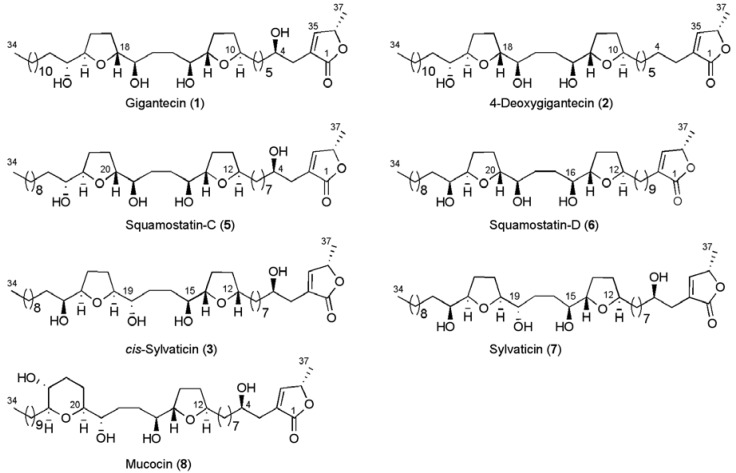
Structures of non-adjacent bis-THF and non-adjacent THF-THP acetogenins.

### 2.1. Total Synthesis of Squamostatin-D *(**6**)*

Squamostatin-D (**6**) is a nonadjacent bis-THF acetogenin isolated from the crushed seeds of *Annona squamosa* L [15]. Fujimoto and co-workers achieved partial structural assignment of the natural product using mass spectrometry and ^13^C NMR spectroscopy. On the basis of NMR data they were able to determine the relative stereochemistry in the non-adjacent bis-THF core, and that both THF rings are *trans* configured. The absolute stereochemistry awaited confirmation by total synthesis.

**Figure 6:**
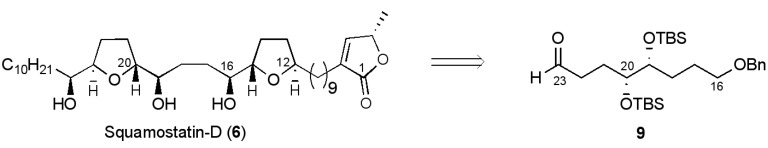
Marshall’s approach to squamostatin-D (**6**).

**Scheme 2:**
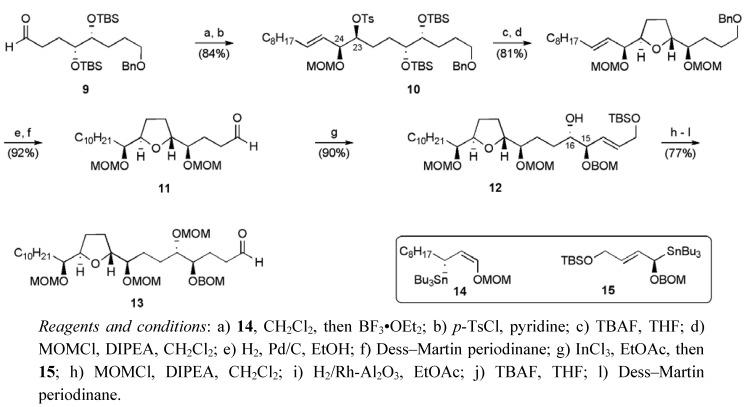
Synthesis of aldehyde **13**.

In 1998 Marshall *et al.* reported the total synthesis of squamostatin-D (**6**) using a linear approach from the known aldehyde **9** (Figure 6) [38]. A key feature in their synthesis was the implementation of highly diastereoselective additions of enantiomerically enriched γ-oxygenated allylic tin and indium reagents to aldehydes (Scheme 2) [39].

**Scheme 3:**
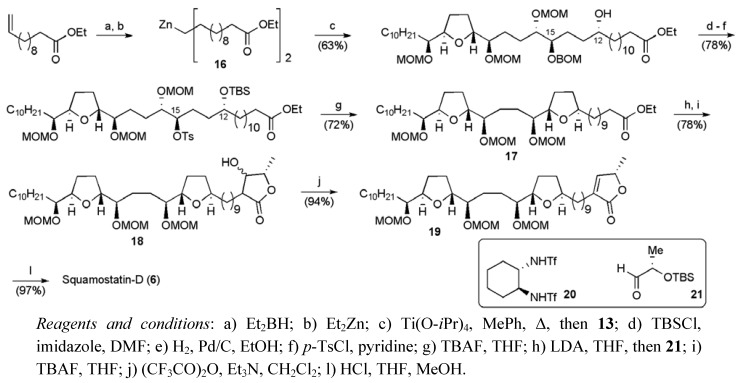
Total synthesis of squamostatin-D (**6**).

The addition allylic tin reagent **14** to aldehyde **8 **was used to set up the (23*R*, 24*R*) *threo* configuration between C23 and C24 in squamostatin-D. The resulting secondary alcohol was converted to tosylate **10**, which cyclized upon exposure to TBAF to afford aldehyde **11** after a series of protecting group and functional group manipulations. The aldehyde **11** underwent an *erythro* selective addition of an γ-oxygenated allylic indium reagent derived from organotin **15**, thereby establishing the 15*R*,16*S* configuration in **12**. The aldehyde **13**, obtained from secondary alcohol **12**, underwent a highly diastereoselective (*dr* > 95:5) addition of the organozinc reagent **16** in the presence of bis-triflamide ligand **20** (Scheme 3). Construction of a fully protected C1-C34 fragment **17** containing the non-adjacent bis-THF core was completed by closure of the second THF ring by displacement of the C15 carbinol.

The butenolide portion was completed through application of chemistry described by Yao *et al* [40]. Deprotonation of ester **17** allowed reaction with aldehyde **21** to give an aldol product, which when desilylated, delivered lactone **18**. Dehydration of lactone **18** was achieved with Tf_2_O and Et_3_N and deprotection of the resulting butenolide **19** completed the synthesis of squamostatin-D (**6**). Squamostatin-D was synthesised in 27 linear steps from 1,4-butandiol in a total yield of 5.5%.

Comparisons between NMR spectroscopic data, optical rotation values and melting points for synthetic and natural materials supported the relative and absolute stereochemistry proposed for squamostatin-D (**6**). Further confirmation of the correct absolute stereochemical assignment was provided through synthesis of the tri-(*R*)-Mosher ester derivative of squamostatin-D, and comparison of ^1^H NMR data with that obtained by Fujimoto’s group for the isolated natural product.

### 2.2. Total Synthesis of 4-Deoxygigantecin (2)

**Figure 7:**
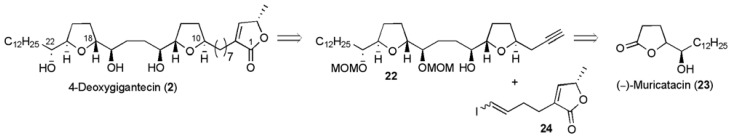
Overview of Makabe’s approach to 4-deoxygigantecin (**2**).

4-Deoxygigantecin (**2**), a structural isomer of squamostatin-D, was isolated by McLaughlin’s group from *Goniothalamus giganteus* [41], and its absolute stereochemistry was not known prior to synthesis. However, the non-adjacent *bis*-THF core stereochemistry was assumed to match that of gigantecin (**1**) which had been assigned by X-ray crystallography [10]. Makabe and co-workers reported a synthesis of 4-deoxygigantecin (**2**) in 1998, which took advantage of a Pd-catalysed cross-coupling to link the THF and butenolide fragments (Figure 7), each of which was assembled in a linear fashion [42,43,44].

**Scheme 4:**
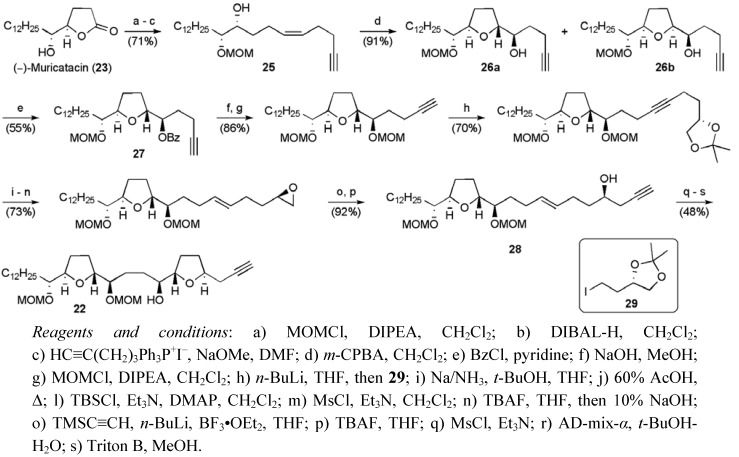
Synthesis of bis-THF alkyne **22**.

*Bis*-THF alkyne **22** was synthesised from *mono*-THF acetogenin derivative (–)-muricatacin (**23**), which is avaliable in 7 steps from propargyl alcohol in 27% yield using the Sharpless asymmetric dihydroxylation to set the C21 and C22 stereochemistry [45]. Protection of (–)-muricatacin (**23**), DIBAL-H reduction and Wittig olefination delivered the *Z*-alkene **25** (Scheme 4). Subsequent epoxidation and cyclisation occurred with little stereoselectivity (**26a**:**26b ~**3:2), and the resulting mixture of tetrahydrofurans was separated following derivatisation. The desired mono-THF diastereoisomer **27** was taken through 10 steps to *E*-alkenol **28**, which was mesylated prior to Sharpless-AD and closure of the second THF under basic conditions. The left hand fragment **22 **was synthesised in 25 steps from propargylic alcohol in 1.9% yield.

**Scheme 5:**
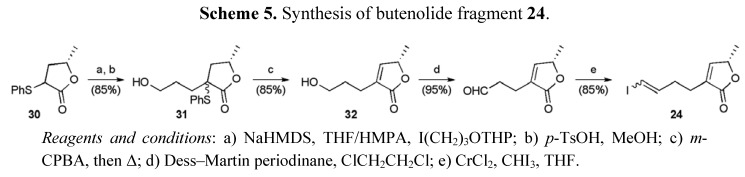
Synthesis of butenolide fragment **24**.

Ethyl (*S*)-lactate served as the source of chirality for the butenolide portion of the molecule, which was prepared in 12 steps and 14.6% yield. An established route provided lactone **30**, which was alkylated to give alcohol **31** (Scheme 5) [46]. Cleavage of the THP group followed by sulfide oxidation and thermal elimination of sulfenic acid returned the butenolide **32** containing a free primary alcohol group that was oxidised and converted to the vinyl iodide **24** using a Takai olefination.

**Scheme 6:**
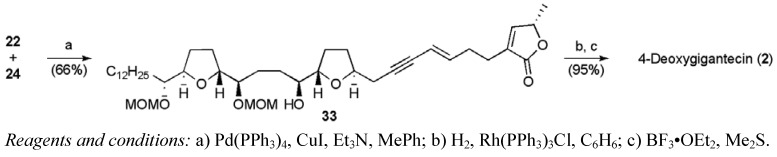
Total synthesis of 4-deoxygigantecin (**2**)

The fragments **22** and **24** could then be combined using Sonogashira chemistry to give enyne **33** (Scheme 6). A selective catalytic hydrogenation followed by global deprotection gave (+)-4-deoxygigantecin (**2**) in 28 linear steps and 1.2% yield from propargylic alcohol. ^1^H NMR and optical rotation were consistent with the data reported for the natural sample of 4-deoxygigantecin (**2**).

### 2.3. Total Syntheses of Gigantecin *(**1**)*

Gigantecin (**1**) is structurally identical to 4-deoxygigantecin, apart from presence of a C4 secondary alcohol possessing the *R* configuration. Interestingly, gigantecin (**1**) has been isolated from two different sources in geographically distinct locations; the bark of *Goniothalamus giganteus* in Southeast Asia and from the seeds of the Brazilian plant *Annona coriacea* [10,47]. Assignment of the gross structure and partial stereochemical determination was initially performed using spectroscopic methods. Subsequently, the structure and stereochemistry of gigantecin was established by X-ray crystallography [10]. Gigantecin (**1**) demonstrated potent cytotoxicity against human tumor cell lines. The cells lines in decreasing activity are U251MG (glioblastoma multiforme) > HT-29 (colon adenocarcinoma) > A-549 (lung carcinoma) > MCF-7 (breast adenocarcinoma). The ED_50 _values were between 4.3–0.003 μg/mL [47]. There have been two total syntheses of gigantecin reported by the groups of Crimmins and Hoye in 2004 and 2006 respectively [48,49].

**Figure 8:**
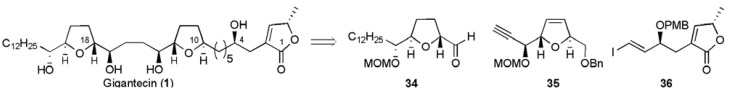
Crimmims’ approach to gigantecin (**1**).

**Scheme 7:**
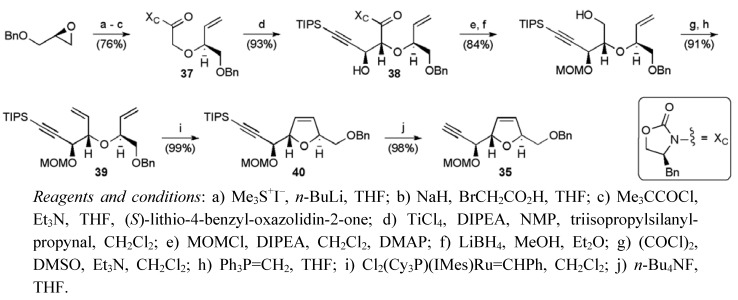
Synthesis of alkyne fragment **35**.

Crimmins’ synthesis is based on a convergent coupling strategy with 3 main fragments **34**, **35** and **36** (Figure 8) [48]. Distinctive features of this route are the use an asymmetric glycolate aldol reaction to assemble acyclic ethers and set the required stereochemistry at C13, C14, C21 and C22 [50], and closure of the five-membered oxygen heterocycles under ring-closing metathesis conditions. The synthesis of the central THF fragment **35** required glycolate **37**, which was derived from (*S*)-benzyl glycidyl ether (Scheme 7). The chiral auxiliary-controlled asymmetric glycolate aldol reaction gave alcohol **38** with high selectivity (>20:1 major:all other isomers).

**Scheme 8:**
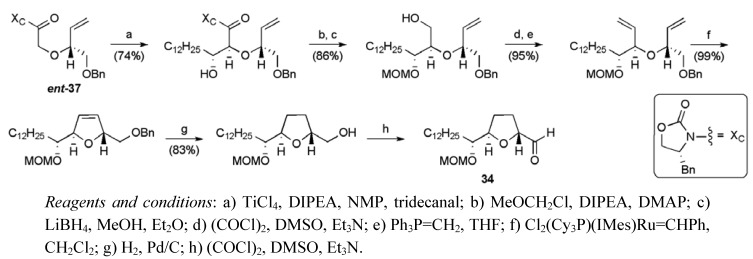
Synthesis of aldehyde fragment **34**.

From aldol **38**, a series of functional group manipulations returned diene **39**, which cyclised in the presence of the second-generation Grubbs catalyst (5 mol %) to give dihydrofuran **40**. Fragment **35 **was obtained by removal of the silyl group, completing its synthesis in 10 steps from (*S*)-benzyl glycidyl ether in 52% yield. The left hand fragment **34** was created in an analogous manner *via* the enantiomeric glycolate ***ent*-37** (Scheme 8), in 11 steps from (*R*)-benzyl glycidyl ether and 38% overall yield.

Auxiliary-controlled diastereoselective alkylation of imide **41** afforded **42** (dr > 98:2, Scheme 9) correctly established the C4 stereogenic centre needed in the butenolide fragment [51]. Reductive cleavage of the *N*-acyloxozolidinone **42**, and subsequent TBS protection of the resulting primary alcohol afforded vinyl bromide **43**. Lithium-bromine exchange and trapping with CO_2_ gave an acrylic acid, which was coupled with secondary alcohol **47** under Mitsunobu conditions to provide diene **44**. The diene was subjected to RCM delivering butenolide **45**. Deprotetion of the primary alcohol, oxidation and Takai olefination secured the desired butenolide fragment **36** in 11 steps from 4-methoxybenzyl alcohol.

Final fragment assembly commenced with the union of the THF fragments **34** and **35** using an asymmetric acetylide addition to afford a single detectable diastereoisomer **48** (Scheme 10). Conversion of benzyl ether **48** to a terminal alkyne **49** then permitted attachment of the butenolide fragment **36** under Sonogashira conditions. Selective hydrogenation of the resulting eneyne **50 **followed by global deprotection provided gigantecin (**1**) in 19 linear steps from benzyl glycidyl ether with a yield of 6.5%.

**Scheme 9:**
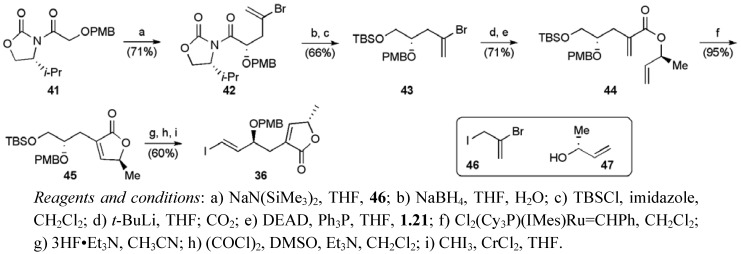
Synthesis of butenolide fragment **36**.

**Scheme 10:**
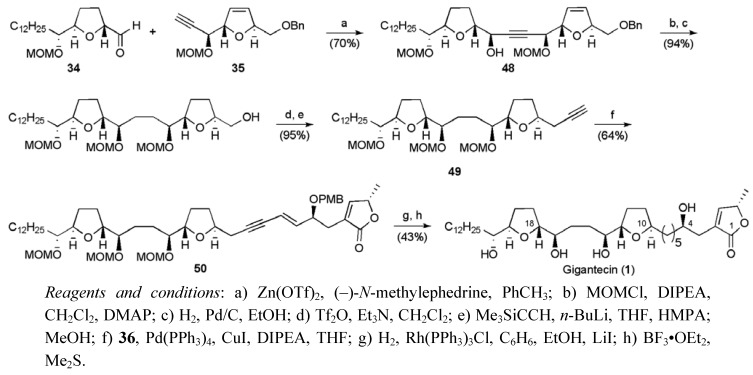
Total synthesis of gigantecin (**1**).

**Figure 9:**
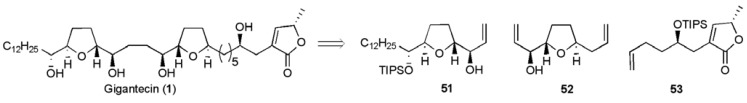
Hoye’s metathesis-based approach to gigantecin (**1**).

Hoye’s approach to gigantecin also exploited a convergent three fragment approach, using a one-pot double metathesis reaction to unite the THF fragments and couple the butenolide portion (Figure 9) [49].

**Scheme 11:**
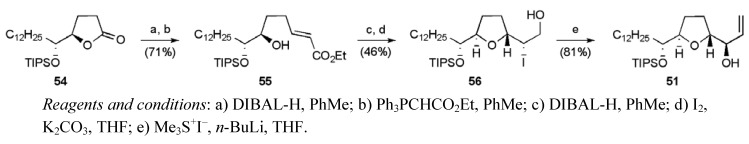
Synthesis of left hand THF fragment **51**.

Both *trans*-THF moieties present within fragments **51 **and **52** were installed by means of selective iodoetherification reactions. The synthesis of left hand fragment **51** proceeded by way of the lactone **54** (4 steps from tridecanal in 73% yield, Scheme 11). DIBAL-H reduction of lactone **54** to the lactol and olefination gave ester **55**. Reduction of **55**, and iodoetherification of the resulting allylic alcohol afforded the iodohydrin as a 4:1 mixture of isomers favouring the desired *trans* THF **56**. Fragment **51** was completed by reaction of the iodohydrin **56 **with dimethylsulfonium methylide. Overall the fragment synthesis required 9 steps from tridecanal and proceeded in 19.3% yield.

**Scheme 12:**
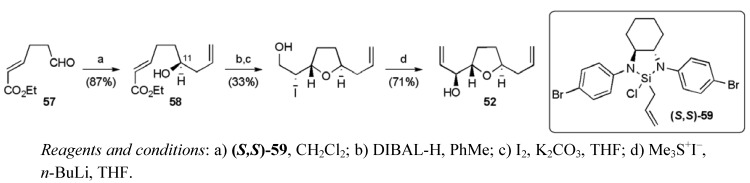
Synthesis of the central THF fragment **52**.

A Leighton asymmetric allylation of aldehyde **57** was employed to introduce the C11 stereogenic centre in the hydroxyester **58** (Scheme 12). The *trans*-THF ring was then created using a similar iodoetherification approach, followed by reaction with the sulfonium ylide to give the second THF fragment **52 **in 7 steps from γ-butyrolactone in a total yield of 9.4%.

**Scheme 13:**
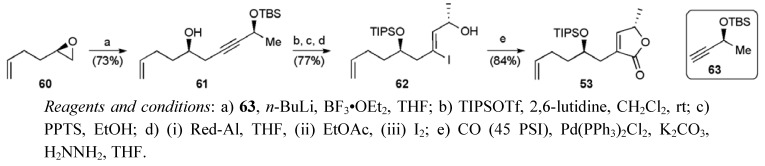
Synthesis of butenolide fragment **53**.

Butenolide fragment **53** was synthesised through a modification of known route (Scheme 13) [52]. Epoxide **60** (available from kinetic resolution of racemic 1,2-epoxy-5-hexene) was opened with the lithiated alkyne **63** to deliver alcohol **61**. After a sequence of protecting group manipulations, hydroalumination and iodine quench gave the vinyl iodide **62**. Palladium-catalysed carbonylative cyclisation was used to create the butenolide and complete the fragment **53** in an overall yield of 21% over six steps from racemic 1,2-epoxy-5-hexene.

The sequencing of the double metathesis reaction was critical and, following tethering of the two allylic alcohol fragments **51** and **52**, the cross-metathesis reaction between the two type-I olefins was best achieved with 4 equivalents of the butenolide fragment under slow addition of Grubbs II catalyst. The cyclisation also proceeded under the same conditions to give the product **65** in 63% yield based on triene **64**. For further discussion of temporary silicon-tethered RCM see Evans’ synthesis of mucocin below. Attempts to perform the RCM first followed by CM led to increased byproduct formation. Selective diene reduction with diimide and global deprotection secured gigantecin (**1**), in a total yield of 4.4% for the longest linear sequence of 13 steps from tridecanal.

**Scheme 14:**
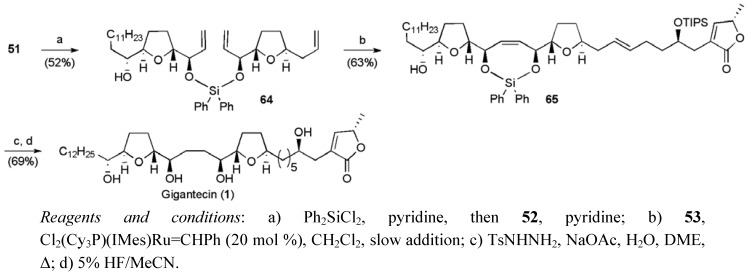
Hoye’s total synthesis of gigantecin (**1**).

### 2.4. Total Synthesis of Squamostatin-C (Bullatanocin) *(**5**)*

Squamostatin-C (**5**) is a structural isomer of gigantecin, that has been isolated from 2 sources; McLaughlin’s group isolated squamostatin-C from from the bark of *Annona bullata* Rich, while Fujimoto’s group isolated squamostatin-C from from the seeds of *Annona squamosa* [15,53]. Relative and absolute structural assignments were performed using mass spectrometry with EI fragmentation and NMR spectroscopy [54,55]. It is of interest that squamostatin-C (**5**) demonstrates potent cytotoxicity against colon cell line HT-29 and lung cell line A-549 with ED_50_ values of less than 10^–8^ g/mL [53].

**Figure 10:**
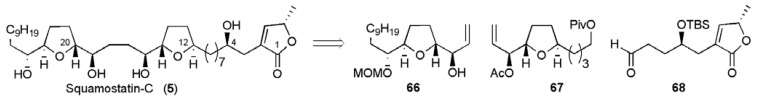
Mootoo’s route to squamostatin-C (**5**) using cross-metathesis to form the non-adjacent bis-THF core.

**Scheme 15:**
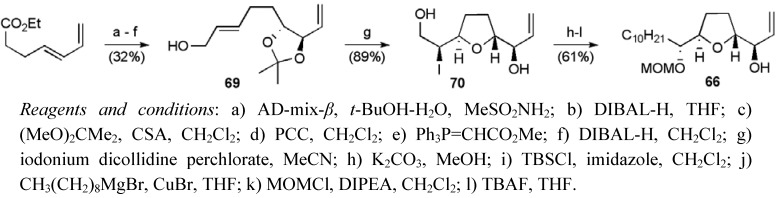
Synthesis of left hand THF fragment **66**.

Mootoo’s synthetic approach to squamostatin-C (**5**) centered on three major fragments (Figure 10) [56,57], which were to be combined by cross-metathesis and Wittig olefination reactions. Both of the *trans*-THF fragments **66** and **67** were created using iodoetherification methodology developed in Mootoo’s laboratory [58]. (*E*)-Ethyl hepta-4,6-dienoate, synthesised by Johnson–Claisen rearrangement of 1,4-pentadien-3-ol, was elaborated through a sequence commencing with regiocontrolled asymmetric dihydroxylation to give the allylic alcohol **69** (ee > 92%, scheme 15) [59]. The alkene **69 **was submitted to iodoetherfication reaction conditions using iodonium dicollidine perchlorate (IDCP) in acetronitrile, affording a single diastereoisomeric *trans*-THF **70**. The ten-carbon side chain was introduced by conversion of the iodohydrin **70** to the epoxide, followed by cuprate addition and protecting group manipulation to give the left hand THF fragment **66**. The overall yield was 12.4% over 13 steps from 1,4-pentadien-3-ol.

**Scheme 16:**
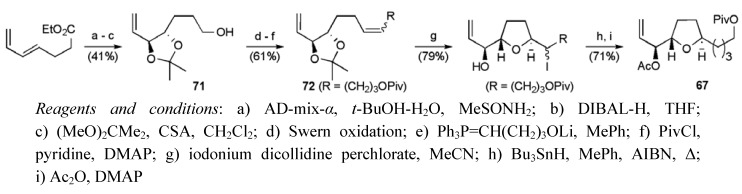
Synthesis of the right hand THF fragment **67**.

The central THF **67** was prepared following a similar route, but switching to AD-mix-α for the selective diene dihydroxylation (Scheme 16). A mixture of alkene isomers **72** (*Z*:*E* 3:1) underwent selective iodoetherification to afford an iodohydrin (*trans*:*cis* dr 11:1), which was deiodonated under radical conditions to secure the fragment **67** in 10.2% yield over 10 steps from 1,4-pentadien-3-ol.

**Scheme 17:**
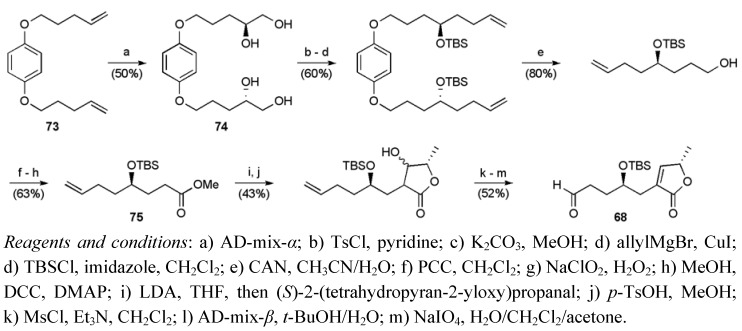
Synthesis of butenolide fragment **68**.

Butenolide **68** was obtained in 14 steps from 6-iodo-1-hexene in a 2.9% yield (Scheme 17). Dihydroxylation of diene **73 **followed by successive recrystallisation afforded tetraol **74** (*S*:*R* 20:1) [60]. The tetraol was converted to ester **75**, which was subsequently elaborated to the butenolide using an aldol approach [61]. Finally, oxidative cleavage of the terminal olefin secured the fragment **68** containing an aldehyde group needed for coupling.

**Scheme 18:**
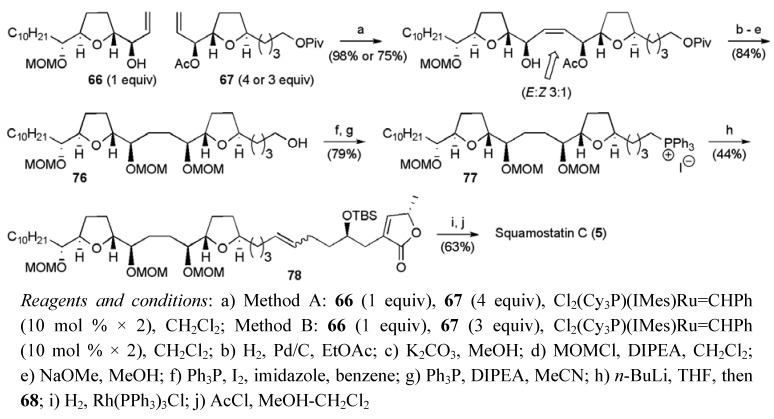
Total synthesis of squamostatin-C (**5**).

Mootoo’s team found that the key CM was most effective when 4:1 and 3:1 ratios of fragments **66 **and **67 **were coupled using Grubbs second-generation catalyst, giving yields of 98% and 75% yields respectively (yields based on limiting alkene, Scheme 18). Alkene hydrogenation and a series of protecting group manipulations gave the primary alcohol **76** that served as a precursor to the phosphonium salt **77**. The fragment coupling using the Wittig reaction proceeded in a moderate yield, but afforded an intermediate that was converted to squamostatin-C (**5**) following selective hydrogenation and global deprotection. Overall the synthesis of squamostatin-C was achieved in 23 steps from (*E*)-ethyl hepta-4,6-dienoate in a total yield 2.2% (based on the limiting fragment).

### 2.5. Total Syntheses of cis-Sylvaticin *(**3**)*

**Figure 11:**
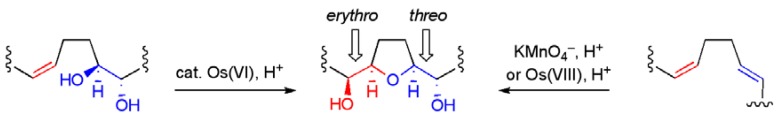
Synthesis of 2,5 *cis*-disubstituted THF diols by metal oxo mediated oxidative cyclisation.

*cis*-Sylvaticin (**3**) was isolated from the leaf extracts of the *Rollinia* mucosa (Jacq.) Baill., and exhibits potent cytotoxicity against A-549 (lung carcinoma) and PACA-2 (pancreatic carcinoma) at nanomolar levels [13]. By contrast with the other non-adjacent bis-THF acetogenins discussed herein, both of the 2,5-disubstituted THF rings in *cis*-sylvaticin (**3**) possess the *cis* configuration. These *cis*-THF systems are ideally suited to synthetic approaches based on oxidative cyclisation of 1,5-dienes by metal oxo species, and both published total syntheses are based on this strategy (Figure 11) [62,63,64,65]. Oxidative cyclisation reactions mediated by osmium and permanganate are stereospecific with respect to the addition of the oxygen functionality across the alkenes, and *trans*-alkenes give rise to *threo* products while *cis*-alkenes lead to *erythro* products [66].

**Figure 12:**
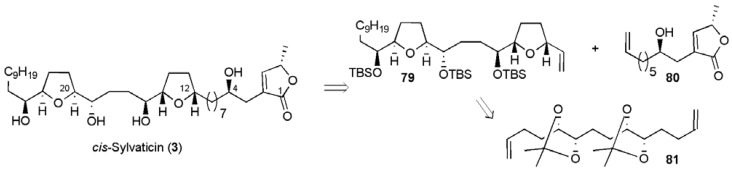
Donohoe’s double oxidative cyclisation approach to *cis*-sylvaticin.

The first report of a total synthesis of **3** came from the Donohoe group in 2006, and made use of an osmium-catalysed double oxidative cyclisation of a protected tetraol **81 **(Figure 12) [62,63,67]. The synthesis of bis-THF fragment **79** began with tetradecatetraene **82**, which is commercially available as a mixture of isomers (*EE*, *EZ*, *ZZ*), or can be separated by chromatography, or was synthesised in three steps from *E,E,E*-cyclododecatetraene.

**Scheme 19:**
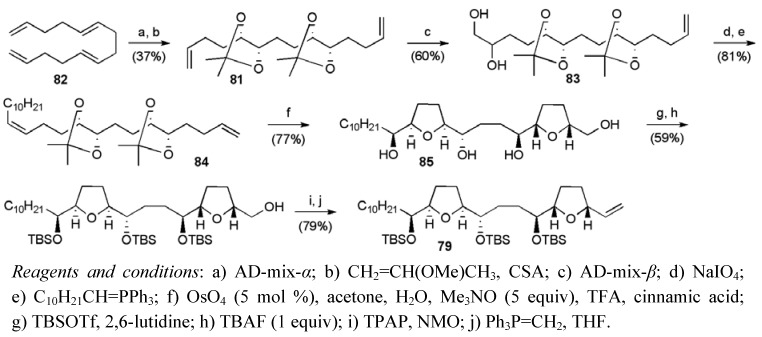
Synthesis of bis-THF fragment **79**.

Asymmetric dihydroxylation of tetraene **82** followed by *in situ* protection selectively gave diene **83 **(ee > 98%, dr > 95:5) in 19% yield from the mixture of isomers or 37% from pure **82** (Scheme 19). The diene **83** was elaborated to introduce the left hand decyl chain into the oxidative cyclisation substrate **84**. Under the acidic conditions of the oxidative cyclisation reaction the acetonide groups were cleaved to reveal the ene-diol systems, which cyclised to afford bis-THF **85** as a single diastereoisomer in high yield. Synthesis of the bis-THF fragment **79** was completed in four further steps, with an overall yield of 6.4% from isomerically pure *E,E*-tetradecatetraene **82 **(10 steps in total).

**Scheme 20:**
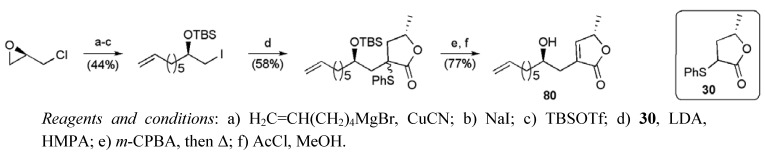
Synthesis of butenolide fragment **80**.

Assembly of the butenolide fragment **80** followed a route described previously by Keum *et al.* (Scheme 20) [68], requiring 6 steps from (*R*)-epichlorohydrin in a total yield of 19.7%. 

**Scheme 21:**
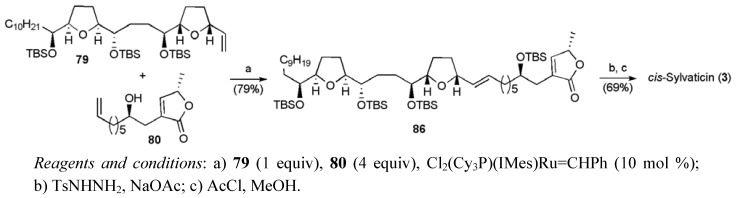
Donohoe’s total synthesis of *cis*-sylvaticin (**3**).

The two alkene fragments were then united by cross-metathesis (Scheme 21), returning the product **86** in 79% yield when excess butenolide fragment (4 equiv) was employed in the presence of Grubbs II catalyst (10 mol %). Diimide reduction and cleavage of the silyl protecting groups completed the synthesis of *cis*-sylvaticin (**3**) in only 13 linear steps from tetradecatetraene **82 **with a total yield of 3.5%.

**Figure 13:**
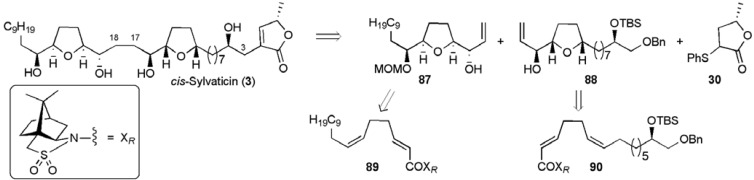
Permanganate-mediated approach to *cis*-sylvaticin (**3**).

**Scheme 22:**
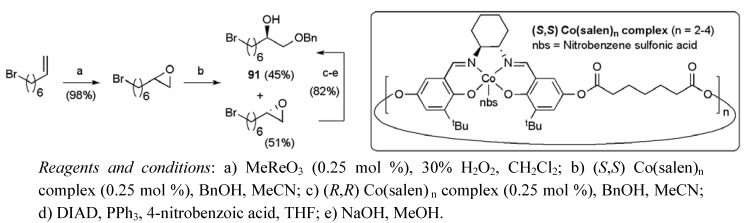
Synthesis of benzyl ether **91**.

A total synthesis of *cis*-sylvaticin has also been achieved in our laboratory using two permanganate promoted oxidative cyclisation reactions of dienes **89** and **90** to introduce 7 of the 9 stereogenic centres present in the natural target (Figure 13) [65]. The more complex diene **90 **required for the C3-C17 fragment **88** was built up from 8-bromooct-1-ene, exploiting a Jacobsen alcoholytic kinetic resolution to correctly establish the C4 alcohol (ee > 99%, Scheme 22) [69].

**Scheme 23:**
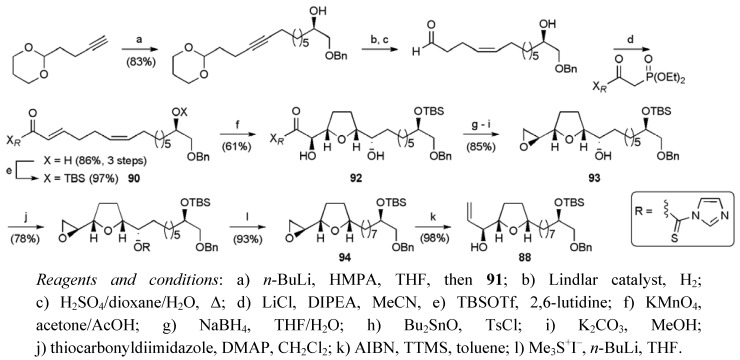
Synthesis of central THF fragment **88**.

The primary alkyl bromide **91** was converted to the *cis*, *trans* 1,5-diene **90** with control over the stereochemistry at both alkenes (Scheme 23). Permanganate oxidative cyclisation then afforded THF diol **92** isolated as a pure diastereoisomer, with diastereocontrol (dr 8.7:1 for the reaction) imparted from the Oppolzer camphorsultam auxiliary [70,71,72,73]. Reductive cleavage of the auxiliary followed by conversion of the resulting vicinal diol to the epoxide **93** enabled selective radical deoxygenation of the secondary alcohol group. Fragment synthesis was completed by treating epoxide **94** with excess Me_2_S=CH_2_, giving allylic alcohol **88 **over 14 steps from 8-bromooct-1-ene in a total yield of 21.7% (17 steps including recycling of (*S*)-epoxide).

**Scheme 24:**
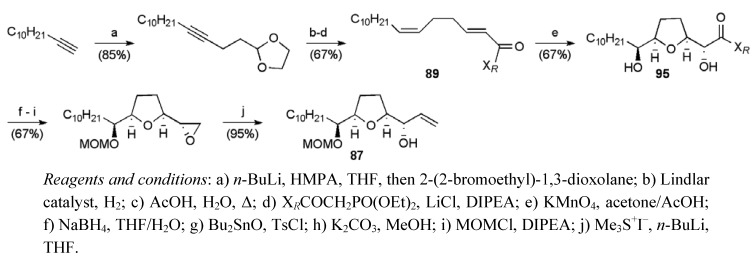
Synthesis of left hand THF fragment **89**.

Similarly, the synthesis of the left hand THF fragment **87** was accomplished in 10 steps from commercially available 1-dodecyne in a total yield of 24.3% (Scheme 24). In the pivotal step, oxidative cyclisation of diene **89** afforded a 9:1 mixture of THF diol diastereoisomers from which the major isomer **95** was isolated in 67% yield [70,71,72,73].

**Scheme 25:**
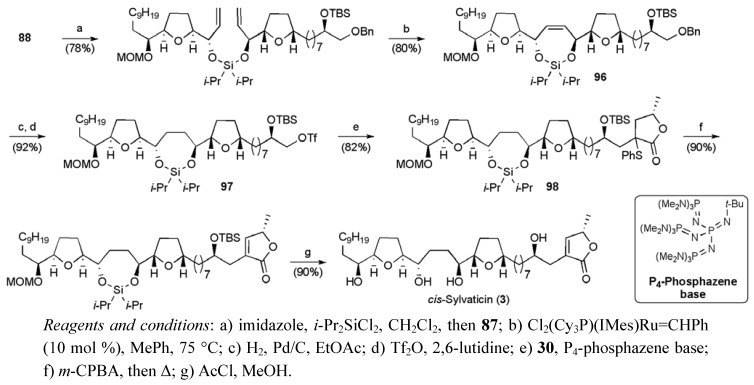
Total synthesis of *cis*-sylvaticin (**3**).

Fragments **87** and **88** were successfully coupled by a di*iso*propylsilyl tethered RCM reaction using second-generation Grubbs catalyst (10 mol %) to give siloxane **96** (Scheme 25). Hydrogenation of the alkene and debenzylation of the RCM product delivered a primary alcohol that was then converted to triflate **97**. Alkylation of lactone **30** was found to proceed efficiently when the P_4_-phosphazene base was used, delivering sulfide **98**. Sulfide **98** was converted to *cis*-sylvaticin (**3**) using the oxidation-sulfoxide elimination and global deprotection sequence described previously. The overall yield for the synthesis was 8.3% for a linear sequence of 21 (24 including inversion of ***ent*-91**) steps from 8-bromooct-1-ene.

### 2.6. Total Synthesis of Sylvaticin *(**7**)*

**Figure 14:**
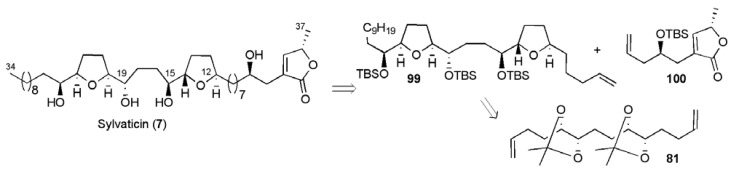
Donohoe’s approach to sylvaticin (**7**).

Sylvaticin (**7**) has been isolated from several Annonaceae sources, and has been co-isolated with its C12 epimer *cis*-sylvaticin (**3**) [13,74,75]. Both compounds exhibit potent cytotoxicity against selected solid human tumour cell lines. A synthesis of sylvaticin was recently disclosed by Donohoe and co-workers, employing the osmium-catalysed oxidative cyclisation to ultimately create both the *cis* and *trans*-disubstituted THF ring systems present in the natural product. The approach was based on their earlier synthesis of *cis*-sylvaticin (Figure 14). However, they needed to convert the central *cis*-THF diol produced from the oxidative cyclisation to the *trans*-hydroxy THF system present in sylvaticin [76].

**Scheme 26:**
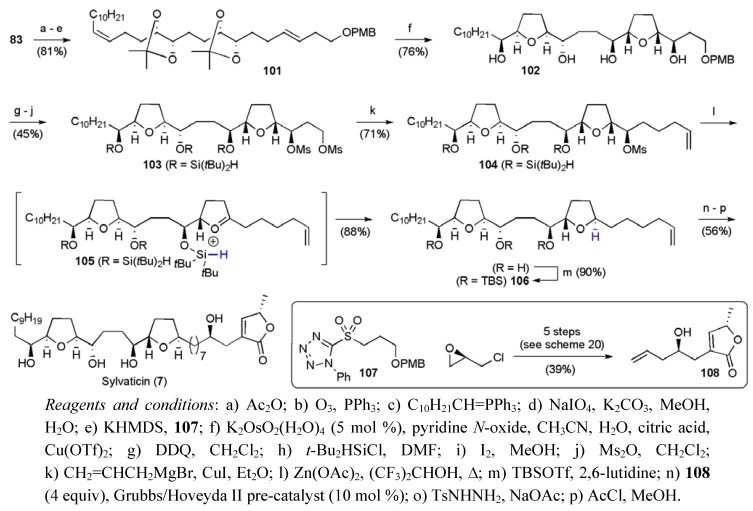
Synthesis of sylvaticin (**7**).

Diol **83**, obtained from mono-dihydroxylation of diene **81** (see Scheme 19) was elaborated in five steps to a new functionalized diene **101** that served as the substrate for an efficient double oxidative cyclisation (Scheme 26). A sequence of selective protecting group manipulations carried out on the oxidative cyclisation product **102** afforded di-mesylate **103**, which was reacted with allyl Grignard in the presence of CuI to provide the substrate **104** for the Lewis-acid promoted hydride shift and intramolecular stereoselective reduction of the intermediate oxa-carbenium **105**. After re-protection of the hydroxyl groups, the terminal alkene **106** was then taken through to sylvaticin using the cross-metathesis coupling strategy described above (see scheme 21), achieving a concise synthesis of the target in 19 linear steps and 2.2% overall yield.

## 3. Total Synthesis of Non-Adjacent THF-THP Acetogenins

**Figure 15:**
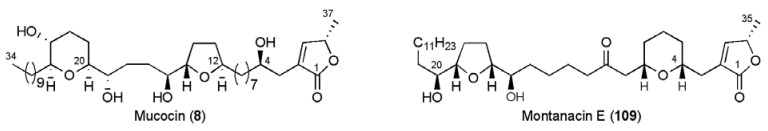
Structures of mucocin (**8**) and montanacin E (**109**).

Only the synthesis of mucocin will be discussed here due to its structural similarity to the non-adjacent bis-THF acetogenins. The total syntheses of montanacin E (**109**) and montanacin D [77,78], which also contain THP and THF rings separated by a carbon chain, will not be reviewed due to the quite different relationship between the cyclic ethers (Figure 15).

### 3.1. Total Syntheses of Mucocin *(**8**)*

**Figure 16:**
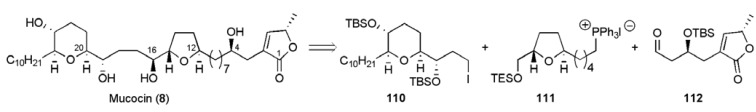
Koert’s synthetic approach to (–)-mucocin (**8**) (1999).

Mucocin (**8**) was the first reported example of an Annonaceous acetogenin that contained a THF ring and an hydroxylated THP ring [79]. Structural assignment was achieved through analysis of MS and NMR data for mucocin, its (16OH,19OH) formylidene acetal derivative, and the corresponding Moshers esters. Mucocin has proved to be a popular target for synthetic chemists, with no fewer than seven total syntheses reported to date. In 1999 Koert’s group disclosed their synthesis of (–)-mucocin [80,81,82]. Their approach required three major fragments **110**, **111** and **112**, which were to be united by Grignard and Wittig reactions (Figure 16). Critically, the stereochemistry at C16 would be set using a chelation controlled Grignard reaction.

**Scheme 27:**
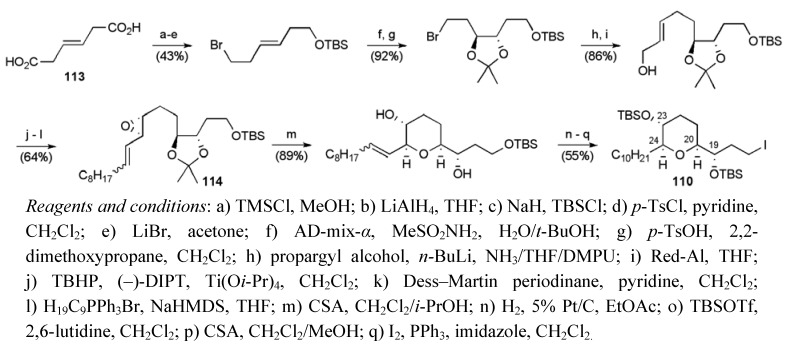
Synthesis of fragment **110**.

The synthesis of the THP fragment **110** began with (*E*)-dihydromuconic acid (**113**) using Sharpless asymmetric epoxidation and dihydroxylation reactions to control the stereochemistry at C19, C20, C23 and C24 (steps f and j scheme 27). Critically, acid deprotection of **114** and the ensuing cyclisation of the resulting epoxydiol favoured 6-*endo* cyclisation over 5-*exo* due to allylic activation of the former pathway, as highlighted previously in studies on 6-*endo* epoxide openings by Nicolaou *et al* [83]. Fragment **110** was synthesised in 17 steps from (*E*)-dihydromuconic acid in 10.8% yield.

**Scheme 28:**
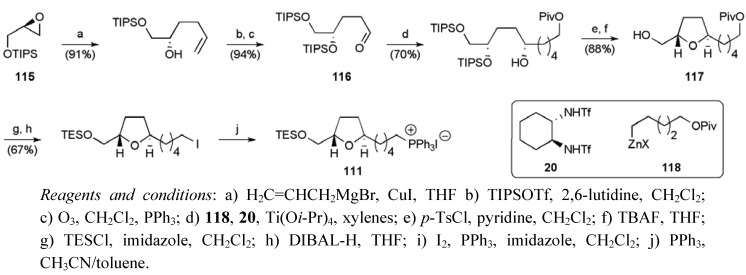
Synthesis of Wittig salt **111**.

The synthesis of the *trans*-THF fragment **111** began with TIPS protected (*R*)-glycidol **115 **(Scheme 28). Key steps included the use of a reagent controlled organozinc addition to aldehyde **116** (dr 95:5) and a Williamson etherification to close the ring in hydroxymethyl THF **117**. Further manipulations returned the Wittig salt **111**, which was used crude in the subsequent olefination reaction.

**Scheme 29:**
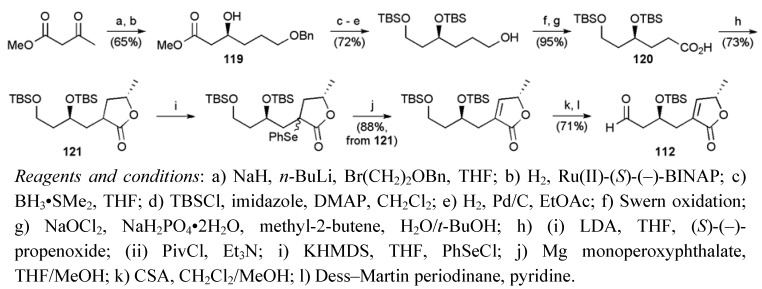
Synthesis of butenolide **112**.

Methyl acetoacetate served as the starting material for the assembly of butenolide fragment **112** (Scheme 29), introducing the C4 stereogenic centre in **119** by enantioselective hydrogenation of the β-ketoester (ee = 96%). The butenolide could then be constructed by alkylation of the dianion of carboxylic acid **120** with (*S*)-(–)-propenoxide, followed by cyclisation to the lactone **121** and dehydrogenation using organoselenium chemistry. 

**Scheme 30:**
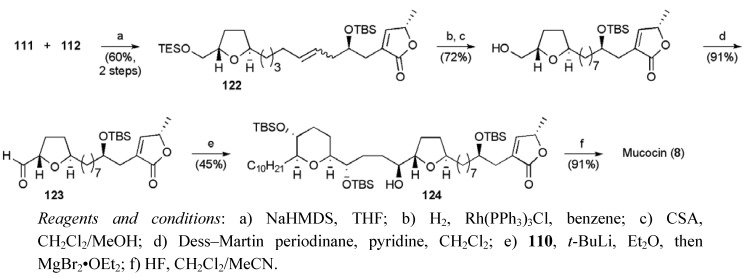
Koert’s total synthesis of mucocin (**8**).

With Wittig salt **111** and aldehyde **112** in hand, coupling mediated by NaHMDS afforded alkene **122** as a mixture isomers, which were converted to the aldehyde **123** (Scheme 30). The aldehyde **123** then underwent a stereoselective addition of the organomagnesium reagent derived from iodide **110** under chelation control (dr 4:1). Desilylation of the resulting alcohol **124 **secured mucocin (**8**) in 19 linear steps from methyl acetoacetate in a total yield of 3.3%.

**Figure 17:**
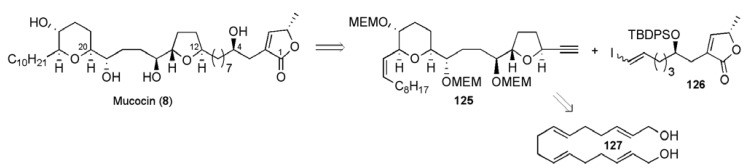
Sinha and Keinan’s route to mucocin (**8**) (1998).

Sinha and Keinan have advanced a strategy for the synthesis of acetogenins, the so-called the “naked alkyl skeleton approach” [84,85], where an unsaturated carbon backbone is synthesised prior to functionalisation by means of stereocontrolled oxidation reactions (e.g. epoxidation, dihydroxylation and oxidative cyclisation). They applied this concept in their synthesis of mucocin, reported in 1998 [86], where the major fragment **125 **was elaborated from a tetraene precursor **127** (Figure 17).

**Scheme 31:**
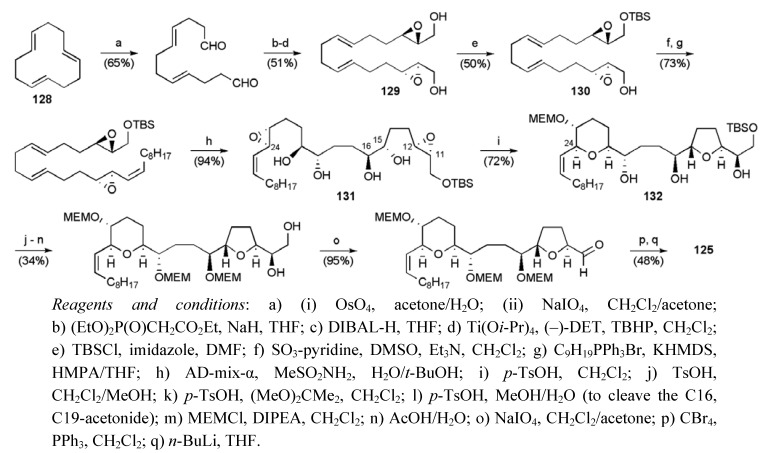
Synthesis of alkyne **125**.

Starting from (*E*,*E*,*E*)-cyclododecatriene (**128**) a two-directional strategy was initially employed to secure the bis-epoxy diol **129** (98% ee), which was desymmetrised by mono-protection to permit the introduction of the alkyl side-chain (Scheme 31). A double Sharpless asymmetric dihydroxylation of both *trans*-alkenes gave tetraol **131**, setting up the molecule for acid-catalysed regioselective cyclisation. As noted above in the Koert synthesis and with precedent in the work of Nicolaou *et al.* [80,81,82,83], cyclisation to the THP **132** was favoured in the left hand epoxy diol system due to allylic activation of C24. By contrast, the C14-C11 epoxydiol system closed onto C12 to give the THF ring, due to a combination of stereoelectronic effects and deactivation of C11 by the adjacent electronegative silylyether group. The synthesis of the fragment **125** was completed in 18 steps from (*E*,*E*,*E*)-cyclododecatriene in 1.3% overall yield.

**Scheme 32:**
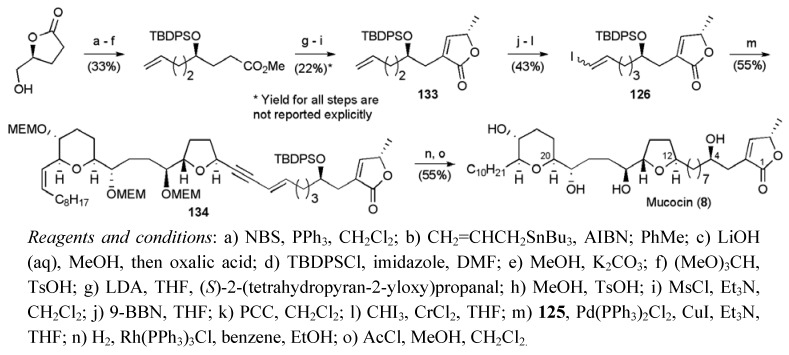
Synthesis of vinyl iodide fragment **126** and total synthesis of mucocin (**8**).

The vinyl iodide fragment **126** was prepared in 12 steps from (*S*)-dihydro-5-(hydroxymethyl)furan-2(3H)-one through application of a route which had previously been published by the same group (Scheme 32) [61]. A Sonogashira coupling reaction between alkyne **125** and vinyl iodide **126** afforded the enyne **134**, which was converted to the natural product following hydrogenation and acidic global deprotection. Mucocin (**8**) was synthesised in a longest linear sequence of 21 steps from (*E*,*E*,*E*)-cyclododecatriene (**128**) in a total yield of 0.4%.

**Figure 18:**
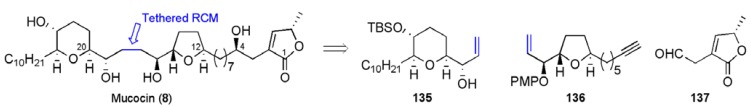
Evans’ route to mucocin (**8**) (2003).

The discovery of efficient olefin metathesis reactions has had a profound influence on synthetic strategy in general, and several groups have applied these powerful transformations in total syntheses of mucocin. The synthetic utility of tethered RCM was recognised by Evans and co-workers in their 2003 synthesis of mucocin as a means to unite the THP and THF containing fragments (Figure 18) [59,87,88,89,90].

**Figure 19:**
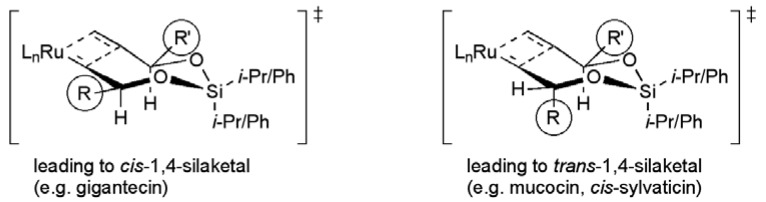
Diastereoselective tethered RCM reactions of silaketals

This was the first reported example of the use of a temporary silicon linkage to assist to selective union of two cyclic ether containing fragments applied within in the synthesis of Annonaceous acetogenins, and built upon earlier studies on diastereoselective RCM reactions [91]. It is noteworthy that the *trans*-disubstituted 1,4-silaketal products were shown to form more slowly under RCM conditions in comparison to the *cis*-isomers in the diastereoselective cyclisation study by Evans (Figure 19). For the total synthesis of mucocin, the less favoured *trans*-1,4-silaketal would be required (compare to the formation of *cis*-silaketal **65 **in Scheme 14, and *trans*-silaketal **96** in Scheme 25).

**Scheme 33:**
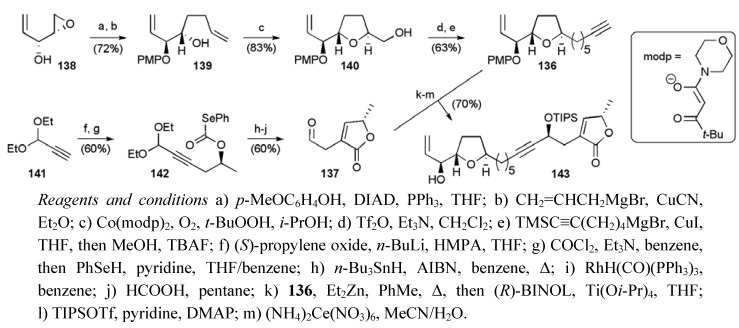
Synthesis of the C1-C17 allylic alcohol **143**.

Epoxy alcohol **138 **(from asymmetric epoxidation of 1,4-pentadien-3-ol in 45% yield) provided a convenient intermediate for the synthesis of the oxidative cyclisation precursor **139** (Scheme 33). Cobalt mediated oxidative cyclisation returned hydroxymethyl THF **140** with excellent *trans* selectivity (dr ≥ 19:1) [92]. Activation of the C11 alcohol in **140** as its triflate derivative permitted introduction of the terminal alkynyl chain using an alkyl cuprate substitution to deliver the central fragment **136**. Their synthesis of the aldehyde fragment **137** started with a regioselective opening of (*S*)-propylene oxide with lithiated alkyne **141**, and subsequent conversion to selenocarbonate **142** (Scheme 33). γ-Lactone formation was achieved under radical conditions, and the resulting *exo*-cyclic alkene isomerised in the presence of a rhodium hydride catalyst to give the butenolide. Acid mediated deprotection gave aldehyde **137** in 36% overall yield for the 5 steps from alkyne **141**. Reaction of the aldehyde **137** with alkyne **136 **proceeded with high diastereoselectivity (dr = 20:1) in the presence of (*R*)-BINOL, and subsequent protecting group manipulations gave allylic alcohol **143** in 8 steps from **138** with a yield of 26%.

**Scheme 34:**
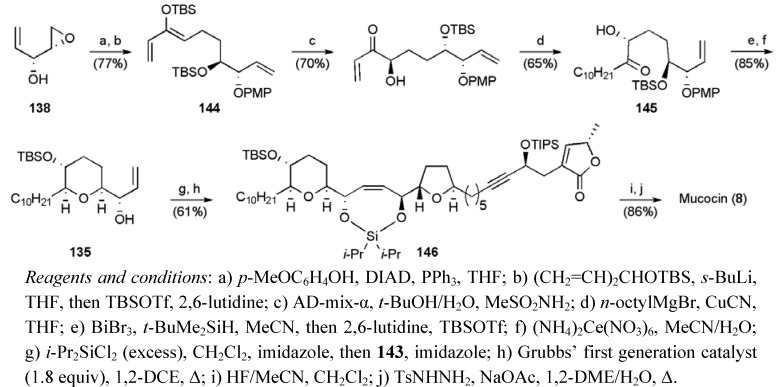
Synthesis of THP fragment **135** and tethered RCM to give mucocin (**8**).

The synthesis of the THP fragment **135** also began with epoxyalcohol **138 **(Scheme 34). A Mitsunobu inversion of alcohol **138** with *p*-methoxyphenol, subsequent regioselective epoxide opening with the lithium homoenolate of TBS protected divinyl alcohol, and *in situ* silylation gave triene **144**. Asymmetric dihydroxylation and introduction of the left hand alkyl chain using a copper-mediated 1,4-addition afforded the ketone **145**, and set the stage for a bismuth-catalysed reductive etherification reaction to deliver the *cis*-THP ring (dr ≥ 19:1) [93,94]. Final protecting group adjustments gave the required THP fragment **135** in 6 steps from **138** with a yield of 30%.

The synthesis was then completed by bringing the fragments **135** and **143** together in a silicon tethered RCM. This tethered coupling reaction required high loadings of the Grubbs’ first generation ruthenium complex (1.8 equiv). Subsequent studies by other groups have shown that lower loadings may be possible using Grubbs II or other catalysts (for examples see scheme 14 and scheme 25). A global desilylation and selective hydrogenolysis gave mucocin (**8**) with a longest linear sequence of 13 steps from 1,4-pentadien-3-ol in 13.6% yield.

**Figure 20:**
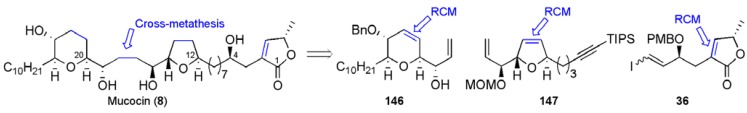
Crimmins’ strategy for the synthesis of mucocin (**8**) (2006).

Crimmins’ approach to mucocin, published in 2006, was also heavily reliant on metathesis in both fragment syntheses and coupling (Figure 20) [95]. Glycolate aldol-RCM chemistry strategies similar to those used in their total synthesis of gigantecin (**1**) were applied in the assembly of the THP and THF fragments **146** and **147**. The butenolide fragment **36** was already available from their earlier total synthesis of gigantecin (**1**) synthesis [48].

**Scheme 35:**
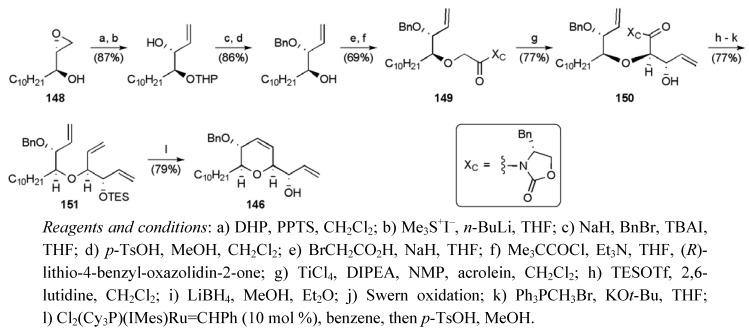
Synthesis of THP fragment **146**.

The synthesis of THP fragment **146** started with known epoxyalcohol **148 **(available in 2 steps from undecanal in 48% yield) (Scheme 35) [96,97]. Glycolate aldol reaction between imide **149** and acrolein provided an excess of the *threo* isomer **150** (dr 11:1), which underwent a series of manipulations to deliver triene **151**. Judicious choice of protecting groups, and preferential closure of the six-membered ring biased the RCM reaction of the triene to favour of the desired dihydropyran, which was desilylated upon acidic work-up to secure the alyllic alcohol **146** in 14 steps from undecanal in a 11.6% yield. 

**Scheme 36:**
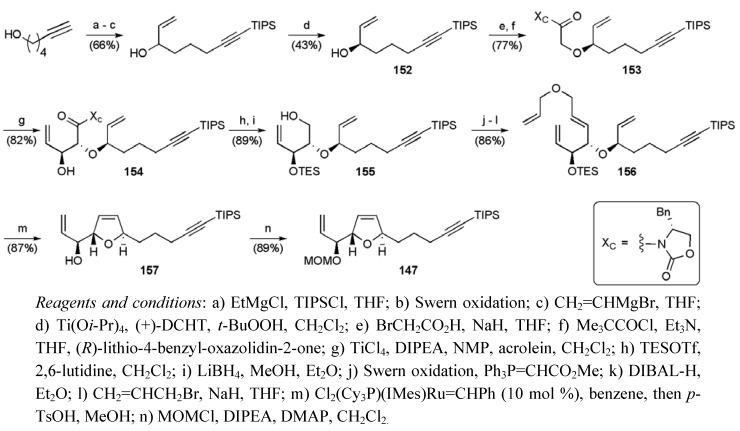
Synthesis of THF fragment **147**.

The substrate for the second glycolate aldol reaction was prepared from the resolved secondary alyllic alcohol **152** (ee 92%, scheme 36). Reaction between imide **153** and acrolein provided *threo*-aldol product **154** with reasonable selectivity (dr 4:1). Initial efforts to close the dihydrofuran using RCM met with some difficulties, which were overcome by implementing Hoye’s “activation” strategy [98]. This required a 3-step conversion of alcohol **155 **to tetraene **156**. With tetraene **156** in hand ruthenium carbene insertion could be controlled and the allylic alcohol **157 **was delivered with good levels of regioselectively (7:1). MOM Protection of alcohol **157** completed the synthesis of the THF fragment in 14 steps in 3.3% overall yield.

**Scheme 37:**
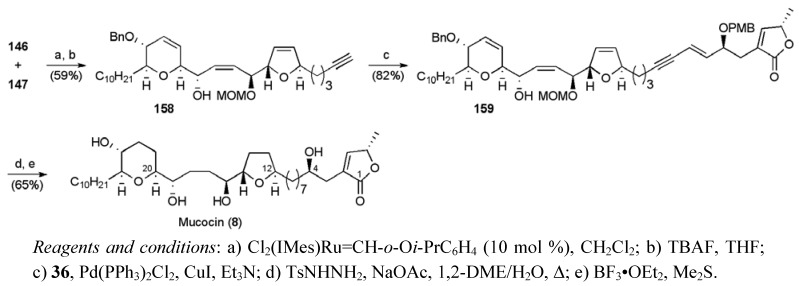
Crimmins’ total synthesis of mucocin (**8**).

The steric hindrance of the MOM ether group apparently deactivated allylic alcohol **147 **towards metathesis relative to allylic alcohol **146**. The differences in reactivity allowed successful CM coupling using Hoveyda-Grubbs catalyst (Scheme 37). The CM product was desilylated, delivering alkyne **158** which was used in a Sonogashira coupling with iodide **36** to secure enyne **149**. Selective hydrogenation and global deprotection gave mucocin (**8**), with a longest linear sequence of 19 steps from alcohol hex-1-yne-6-ol in 1.0% yield.

**Figure 21:**
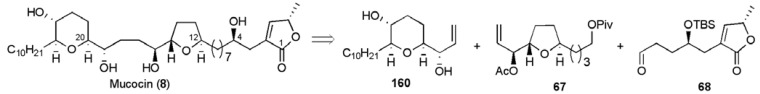
CM and olefination approach to mucocin (**8**) used by Mootoo and co-workers (2006).

Metathesis was also pivotal in Mootoo’s route to mucocin reported in 2006, exploiting CM and olefination reactions to assemble the key fragments paralleling their earlier synthesis of squamostatin-C (Figure 21). Due to the fact that the C1-C18 segments of mucocin and squamostatin C are identical, Mootoo was able to utilize the same C1-C7 and C8-C17 fragments **68** and **67** (see scheme 16 and scheme 17) [57,99].

**Scheme 38:**
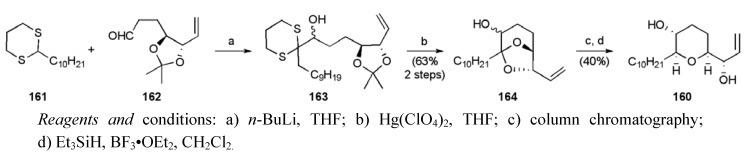
Synthesis of THP fragment **160**.

The synthesis of the THP fragment **160 **began with aldehyde **162**, obtained from Swern oxidation of **71** (Scheme 38) [57]. Addition of lithiated dithiane **161** gave secondary alcohol **163 **as an epimeric mixture. Exposure of **163** to mercury (II) perchlorate induced acetal exchange, which allowed the alcohol epimers **164** to be separated using column chromatography. Reductive acetal cleavage furnished the desired THP fragment **160** in 8 steps from 1,4-pentadien-3-ol and 6.7% yield. 

**Scheme 39:**
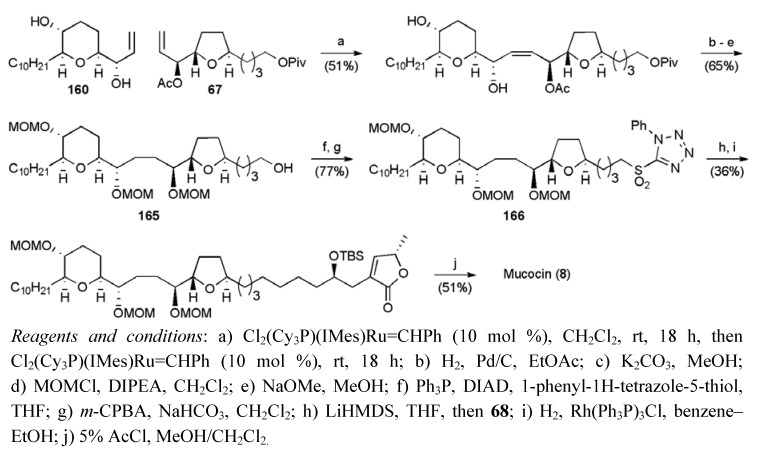
Mootoo’s total synthesis of mucocin (**8**).

Cross-metathesis of allylic alcohol fragment **160** and a three-fold excess of acetate **67** in the presence of Grubbs II catalyst afforded the coupled product in 51% yield (Scheme 39). Hydrogenation and protecting group exchange delivered alcohol **165**, which was converted to sulfone **166** to enable union with the aldehyde fragment **68** under a Julia-Kocienski olefination conditions. Selective hydrogenation and global deprotection completed the synthesis of mucocin (**8**), with a longest linear sequence of 20 steps from 1,4-pentadien-3-ol in 0.5% yield.

**Figure 22:**
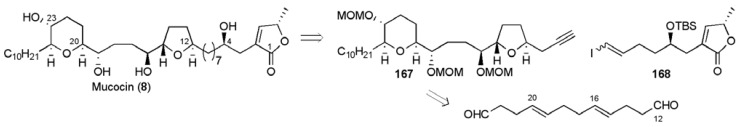
Nakata’s retrosynthetic analysis of mucocin (**8**) (2002).

Takahashi and Nakata published two distinct total syntheses of mucocin both in 2002. In the first reported approach the natural product was assembled from two major fragments, the more complex bis-cyclic ether fragment **167** deriving from (4*E*,8*E*)-dodeca-4,8-dienedial (Figure 22) [100].

**Scheme 40:**
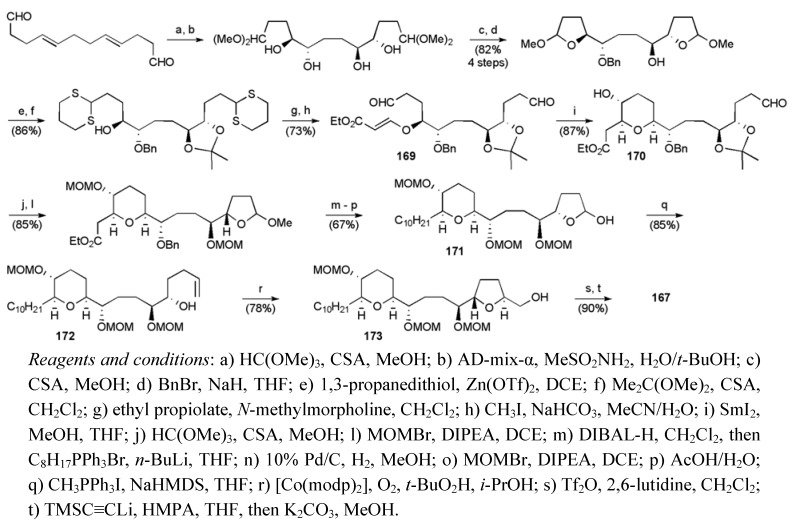
Synthesis of alkyne **167**.

Initial two-directional elaboration of (4*E*,8*E*)-dodeca-4,8-dienedial (available in 2 steps from (*E*,*E*,*E*)-cyclododecatriene, see scheme 31) provided a bis-acetal that was desymmetrised by mono-benzylation (Scheme 40). Further manipulations led to α,β-unsaturated ester **169**, which underwent reductive cyclisation mediated by SmI_2_ to install the *cis*-THP ring in **170** [101]. Protecting group adjustments gave lactol **171**, then Wittig olefination secured the alkenol **172**. Nakata *et al.* employed the *trans*-selective cobalt oxidative cyclisation, also seen in the Evans synthesis of mucocin (see Scheme 33), to create the THF ring in alcohol **173** with high selectivity [92]. Conversion of alcohol **173** to its triflate and subsequent displacement with lithiated TMS acetylene followed by *in situ* desilylation gave fragment **167** in 20 linear steps from (*E*,*E*,*E*)-cyclododecatriene with an overall yield of 9.9%.

**Scheme 41:**
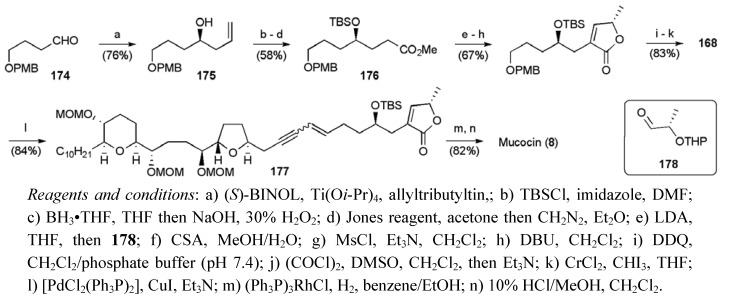
Synthesis of vinyl iodide **168** and total synthesis of **8**.

During the synthesis of the butenolide fragment **168**, the stereochemistry of the C4 carbinol was controlled using an asymmetric allylation (ee > 98%, scheme 41). Subsequent conversion of the allylation product **175** to the ester **176** permitted the creation of the butenolide system using an aldol-lactonisation-dehydration sequence. Cleavage of the electron-rich PMB group, oxidation of the resulting primary alcohol and Takai olefination delivered the required fragment **168** in 13 steps from 1,4-butandiol in a total yield of 21.1%.

The fragments **167** and **168** were combined through the well established Sonogashira coupling strategy to give enyne **177** in good yield, which following selective hydrogenation over Wilkinson’s catalyst and global deprotection afforded mucocin (**8**), with a longest linear sequence of 23 steps from (*E*,*E*,*E*)-cyclododecatriene and corresponding 6.8% yield.

**Figure 23:**
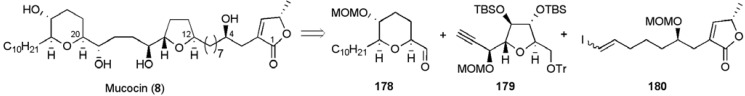
Synthesis of mucocin (**8**) from carbohydrate starting materials (2002).

**Scheme 42:**
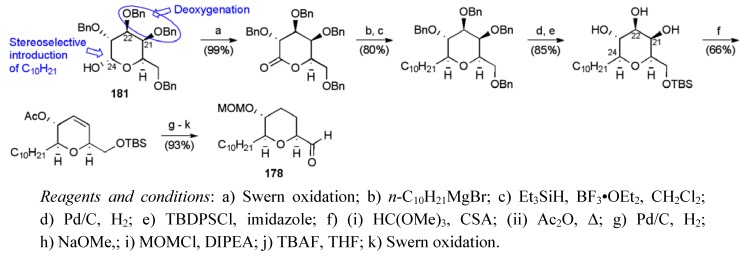
Synthesis of THP fragment **178**.

Takahashi and Nakata published an alternative route to mucocin, wherein each of the three major fragments **178**-**180** was constructed from carbohydrate starting materials (Figure 23) [102,103,104]. Synthesis of THP fragment **178** began with commercially available benzyl ether **181**, deoxygenating at C21/22, and introducing the *n*-decyl side-chain through a Grignard addition-oxo-carbenium ion reduction sequence (Scheme 42). THP fragment **178** was synthesised in 12 steps from benzyl ether **181 **in 41.3% yield.

**Scheme 43:**
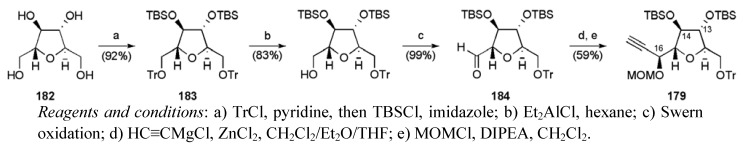
Synthesis of THF fragment **179**.

Commercially available 2,5-anhydro-D-mannitol (**182**) served as the starting material for the THF containing fragment **179** (Scheme 43). Desymmetrisation of a fully protected intermediate **183** afforded the opportunity to create the C16 carbinol stereogenic centre by means of a stereoselective acetylide addition to aldehyde **184 **(dr = 93:7). Protection of the resulting hydroxyl group as its MOM ether gave the required fragment **179** in a total of 5 steps from 2,5-anhydro-D-mannitol in 44.6% yield, but requiring later deoxygenation at C13/14.

**Scheme 44:**
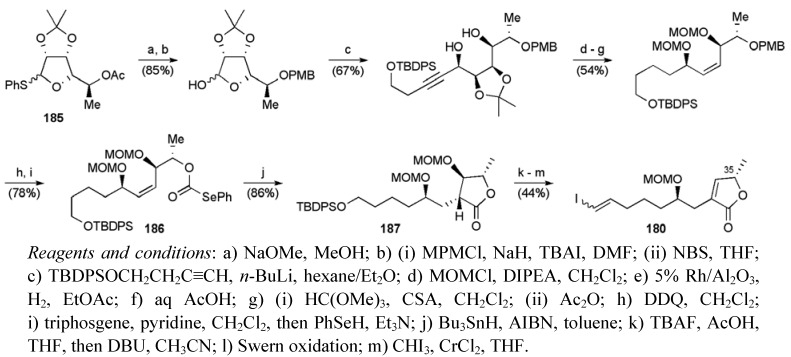
Synthesis of butenolide fragment **180**.

The butenolide fragment **180 **derived from L-rhamnose derivative **185** (3 steps from L-rhamnose) (Scheme 44). It is of note that only the C5 carbinol stereogenic centre present in the deoxy sugar was translated directly into mucocin (corresponds to C35 in mucocin). Thioacetal **185** was converted to the selenocarbonate **186**, which was the substrate for an acyl radical cyclisation to afford lactone **187**. Elimination with DBU followed by silyl deprotection, oxidation and Takai olefination delivered the butenolide **180** in 18 steps from L-rhamnose.

**Scheme 45:**
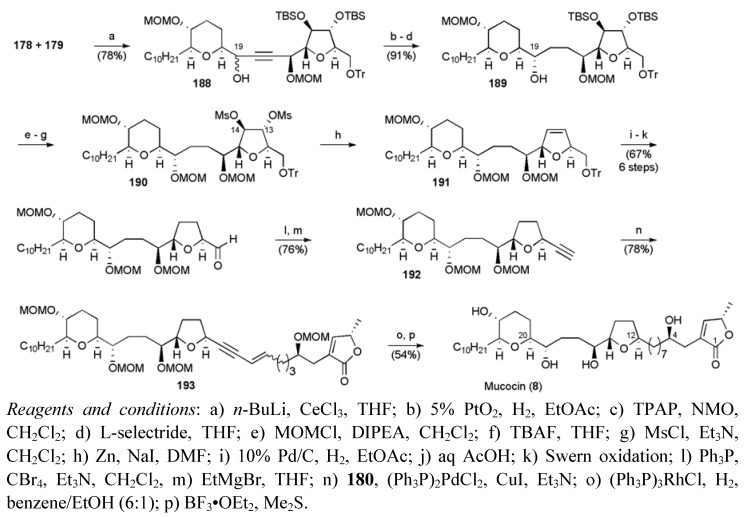
Takahashi’s synthesis of mucocin (**8**).

The THF and THP fragments were united by reacting lithiated alkyne **179 **with aldehyde **178**, giving a mixture of epimers **188** favouring the undesired diastereomer (Scheme 45). Alkyne reduction, then alcohol oxidation followed by stereoselective hydride reduction corrected the C19 carbinol stereochemistry in **189**. The final vicinal deoxygenation at C13/14 was carried out from reductive elimination of the di-mesylate **190**, followed by hydrogenation of the resulting dihydrofuran **191**. Further transformations to the alkyne **192** enabled application of the Sonogashira cross-coupling methodology to attach the butenolide fragment **180**. Finally, selective hydrogenation and global deprotection of enyne **193 **gave mucocin (**8**) in 28 linear steps from benzyl ether **181 **and a corresponding yield of 6.3%.

## 4. Summary

A wide variety of synthetic methodologies have been applied to the synthesis of non-adjacent bis-THF and THF-THP acetogenins. However, some more broadly applicable strategies have emerged. Epoxides and diols have been key intermediates in many of the approaches discussed, taking advantage of well-established catalytic transformations including the Sharpless asymmetric epoxidation and dihydroxylation reactions. Reagent controlled asymmetric C-C bond-forming methodologies such as the glycolate aldol, and the additions of γ-oxygenated allyl metal reagents and organozincs to aldehydes have been used to correctly establish vicinal oxygen stereochemistry. Cyclic ether formation (THF and THP) has most commonly been achieved *via* intramolecular epoxide opening reactions or by displacement of sulfonate leaving groups. Highly stereoselective syntheses of *trans*-THFs have been carried out through iodoetherification and Co-catalysed oxidative cyclisations of bis-homoallylic alcohols and acetals, while metal-oxo mediated oxidative cyclisations have become reliable reactions for the synthesis of *cis­*-THF diols from dienes and dihydroxyalkenes. Subsequently, methodology to transform *cis­*-THF diols into *trans*-hydroxyalkyl THFs was applied to the synthesis of sylvaticin. For the synthesis of the 2,6-*cis*-disubstituted THP system found in mucocin, reductive etherification, and SmI_2_ reductive cyclisation of alkenals have also proved to be highly stereoselective.

In terms of assembling the carbon framework of the non-adjacent bis-THF and THF-THP, two broad strategies can be considered; coupling three major heterocyclic fragments, or approaches where the carbon backbone (or a substantial part of it) is in place prior to forming the cyclic ether core structures. An attractive aspect of the later approach is that the carbon framework can be brought together using robust C-C bond-forming reactions such as olefination. However, the subsequent selective transformation of polyenes need to be controlled. Sonogashira coupling has been used extensively as a reliable means to connect the butenolide system to the pre-formed bis-cyclic ether core structures. Other fragment coupling methodologies have included asymmetric additions of acetylides and alkyl metal reagents to aldehydes, and olefination reactions. Metathesis has also emerged as a powerful fragment coupling method, either directly through cross-metathesis, or by the use of a temporary tether. A limitation of the former method has been the requirement for an excess of one of the fragments, although the recovered fragment can be recycled in some cases. In addition, ring-closing metathesis has also been exploited as a method to form the THF and THP ring systems.

It is apparent from the examples discussed above that the groups of *Annonaceous* acetogenins containing non-adjacent bis-cyclic ether cores have attracted substantial interest from synthetic chemists. This interest has derived, in part, due to the potent biological activity of these acetogenins. However, it can be seen from the diversity of chemistries employed that these non-adjacent systems have also stimulated and inspired the development of synthetic methodology and are likely to continue to do so for some time to come.
